# The Microbiome as a Key Regulator of Female Genital Tract Barrier Function

**DOI:** 10.3389/fcimb.2021.790627

**Published:** 2021-12-17

**Authors:** Andrew Plesniarski, Abu Bakar Siddik, Ruey-Chyi Su

**Affiliations:** ^1^ JC Wilt Infectious Diseases Research Centre, National Microbiology Laboratories, Public Health Agency of Canada, Winnipeg, MB, Canada; ^2^ Department of Medical Microbiology and Infectious Diseases, Faculty of Health Sciences, University of Manitoba, Winnipeg, MB, Canada

**Keywords:** microbial factors, host factors, microbiome, barrier, vagina, female genital tract (FGT), tissue explant, sexually transmitted infection (STI)

## Abstract

The microbiome, the collection of microbial species at a site or compartment, has been an underappreciated realm of human health up until the last decade. Mounting evidence suggests the microbiome has a critical role in regulating the female genital tract (FGT) mucosa’s function as a barrier against sexually transmitted infections (STIs) and pathogens. In this review, we provide the most recent experimental systems and studies for analyzing the interplay between the microbiome and host cells and soluble factors with an influence on barrier function. Key components, such as microbial diversity, soluble factors secreted by host and microbe, as well as host immune system, all contribute to both the physical and immunologic aspects of the FGT mucosal barrier. Current gaps in what is known about the effects of the microbiome on FGT mucosal barrier function are compared and contrasted with the literature of the gut and respiratory mucosa. This review article presents evidence supporting that the vaginal microbiome, directly and indirectly, contributes to how well the FGT protects against infection.

## The Female Genital Tract: Anatomy and Microbiome

**Figure 1:**
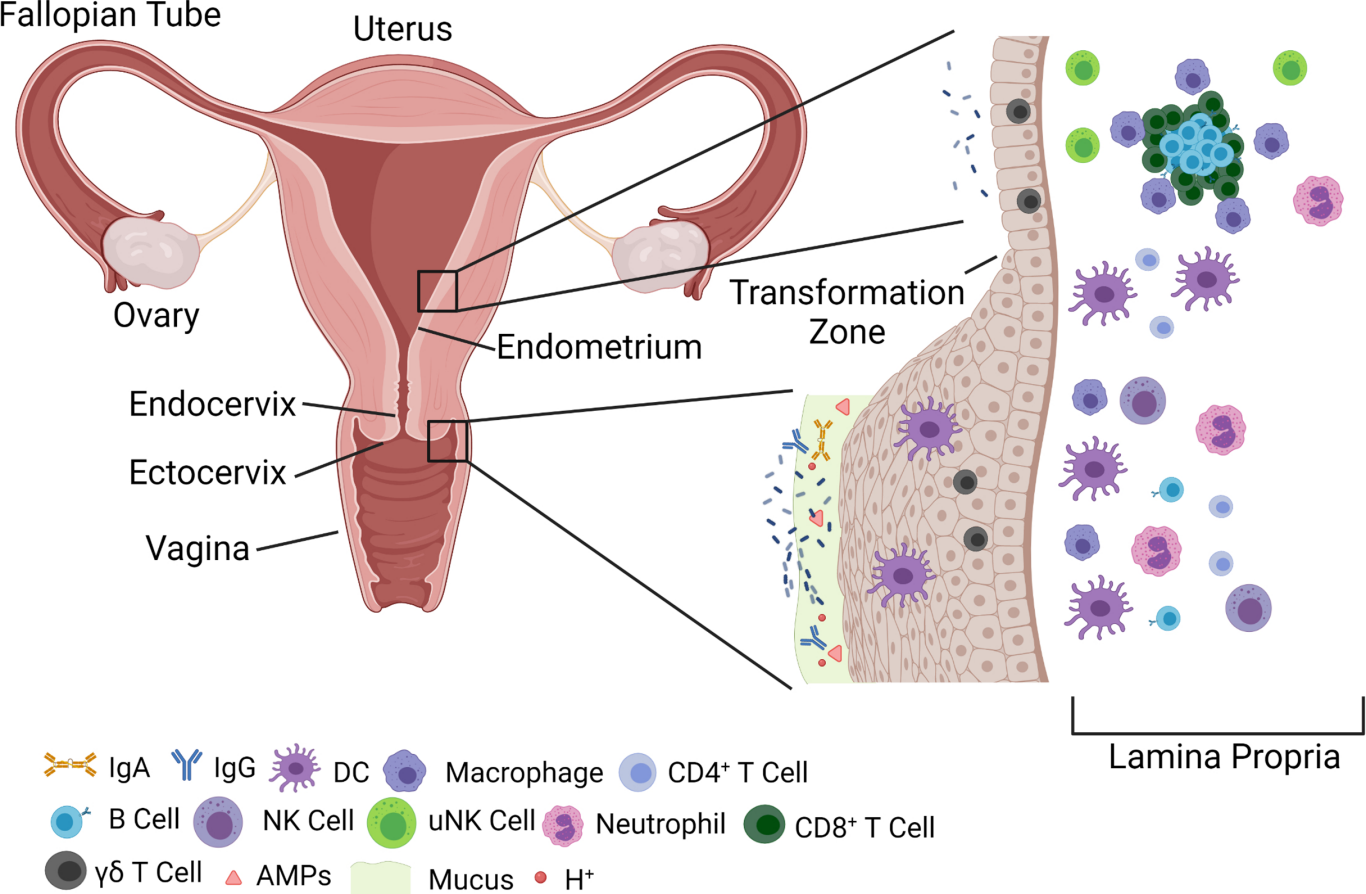
Overview of FGT anatomy and components of barrier function. The FGT is divided generally into an upper (endometrium and endocervix) and a lower (vagina and ectocervix) tract. The lower FGT is considered the primary point of contact for pathogens, and harbors multiple innate immune mechanisms, such as AMPs, mucus, and immunoglobulins that prevent transmission across the epithelial barrier. The epithelium itself is composed of a stratified squamous epithelium that arises from a basement membrane and terminates in fully keratinized, senescent, cells. Microbiota, primarily *Lactobacillus*, can be found in high abundance within the lower FGT, but have also been shown to be resident within the upper FGT in lower numbers. As the epithelium progresses to the upper FGT it changes to a single columnar epithelium at a junction known as the ‘transformation zone’ between the ectocervix and endocervix. The upper FGT harbors unique uNK populations, as well as lymphoid aggregates consisting of B cells surrounded by CD8^+^ T cells and macrophage. These immune gatekeepers balance immune surveillance and response with the need to maintain a fertile environment for pregnancy. FGT, Female genital tract; IgA, Immunoglobulin A; IgG, Immunoglobulin G; DC, Dendritic cell; NK, Natural killer; uNK, Uterine natural killer; AMP, Antimicrobial peptide.

The female genital tract (FGT) can be divided into two distinct regions: lower and upper. The lower part of the FGT is composed of the vaginal canal and ectocervix, while the upper region is defined by the endocervix and uterus proper (Carias and Hope, 2018). The epithelium of the vaginal canal is organized in a stratified squamous configuration up until and including the ectocervix, which enables a barrier with multiple layers of epithelial cells to protect against extracellular pathogens (**Figure 1**). The stratified squamous epithelium originates from a basement membrane lined with progenitor cells, which then mature into fully senescent, and then keratinized epithelium (Chung et al., 2019; Ali et al., 2020). This allows for multiple levels of tight junction formation that keep out pathogens, while allowing the layer closest to the lumen to still be permissive to transudate from the blood and slough off to allow for mucosal shedding. This stratified squamous layer transitions to a simple columnar epithelial pattern at a point known as the ‘transformation zone’, which defines the area where the ectocervix becomes the endocervix. The simple columnar epithelial barrier continues into the uterine endometrium and beyond, and, as a result, the upper FGT has historically been thought to be more vulnerable to pathogens (Shattock and Moore, 2003). This is relevant in cases of cervical ectopy, more common among women who are young, pregnant, or on oral contraceptives, where the endocervix protrudes into the vaginal canal (Loudon et al., 1978; Wright et al., 2014). This protruding endocervix increases the vulnerability of the upper FGT to infection during sexual intercourse, and has been associated with the acquisition of sexually transmitted infections (STIs), such as in the case of chlamydia or human immunodeficiency virus (HIV) (Lee et al., 2006; Kleppa et al., 2015). In addition to the physical barrier of the epithelium, there is a layer of cervicovaginal mucus composed of mucus secreted by goblet cells of the endocervix that combines with cellular debris, vaginal transudate, and immune components to create a matrix that traps potential pathogens and prevents them from reaching or interacting with the epithelial layer and potential target cells (Lacroix et al., 2020). These immune components include secreted proteins such as immunoglobulins, which can bind to pathogens and interact with the cervicovaginal matrix to allow clearance from the FGT by mucus flow (Jensen et al., 2019), antimicrobial peptides (AMPs), and proteases. Despite being a mucosal membrane, where IgA antibodies tend to predominate, the vaginal mucosa is primarily characterized by IgG antibodies that are thought to originate from the vaginal transudate produced from the plasma that crosses from the bloodstream into the vaginal canal (Fahrbach et al., 2013; Oh et al., 2019). In addition to physical methods, there are chemical methods of pathogen protection that rely on the maintenance of an acidic environment within the lower FGT. This low pH environment feeds into, and in turn is fed by, a *Lactobacillus* dominant microbiome (Linhares et al., 2011).

The microbiome of the FGT differs between the lower and upper portions of the tract, with the lower FGT estimated to have a bacterial load of 10^2^-10^4^ fold higher than that seen in the upper FGT (Chen et al., 2017; Baker et al., 2018). Microbiomes of the lower FGT, henceforth referred to as vaginal microbiomes, are divided into five separate community state types (CSTs) that capture the overall dominance of a particular bacterium within the vagina (Ravel et al., 2011; Ma and Li, 2017). While bacteria of each state are all considered commensal, or regular residents in healthy individuals, microbiomes that are non-*Lactobacillus* dominant tend to be associated with poor health outcomes and high degrees of inflammation (Gautam et al., 2015; Petrova et al., 2015; Ma and Li, 2017; De Seta et al., 2019). The vaginal mucosal microbiome is typically less diverse than other sites, with *Lactobacillus* species comprising the major microbiota of the vaginal mucosa (Ravel et al., 2011; Aldunate et al., 2015; Petrova et al., 2015; Song et al., 2020). Among the *Lactobacillus* species, *Lactobacillus crispatus*, *L. gasseri*, *L. iners*, and *L. jensenii* are the most common species identified at the human vaginal mucosa. A large-scale cross-sectional study showed that *L. crispatus, L. gasseri, L. iners, and L. jensenii* were dominant in 26.2%, 6.3%, 34.1% and 5.3% of the samples analyzed, respectively (Ma et al., 2012). The remaining 28% of samples were dominated with non*-Lactobacillus* species and displayed high diversity in microbial composition (Ma et al., 2012). Women are grouped into the five CSTs based primarily on the predominance of a given *Lactobacillus* species in the vaginal mucosa. CST-I, -II, -III, and -V are characterized by *L. crispatus*, *L. gasseri*, *L. iners*, and *L. jensenii* dominance, respectively, whereas CST-IV represents a highly diverse, or non*-Lactobacillus* dominant, microbiome (Ravel et al., 2011). CST-I, -II, and -V are considered optimal vaginal microbiomes, characterized by low pH (pH less than 4.0–5.0), high lactic acid concentrations, and a less inflammatory state of the vaginal microenvironment (Hedges et al., 2006; Ravel et al., 2011). CST-III women who are dominated by *L. iners* differ from other *Lactobacillus* dominated CST groups in that they display higher pH ranges and relatively higher inflammatory statuses at the vaginal mucosa (Verstraelen et al., 2009). Ongoing studies found differences in the genome size and genome structure of *L. iners* compared to other *Lactobacillus* species, however additional studies are required to explain how these differences contribute to the differences in vaginal milieu (Mendes-Soares et al., 2014). CST-IV is dominated by a varied range of facultative or strictly anaerobic bacteria in the vaginal mucosa, and is considered a non-optimal vaginal microbiome. It is characterized by higher pH levels (pH >5.0), lower levels of lactic acid, and an increased inflammatory state (Hedges et al., 2006; Ravel et al., 2011; Vagios and Mitchell, 2021). Despite the differences displayed in CST-IV, when present without any symptoms it is considered a healthy state. Bacterial genera that dominate in the CST-IV group, however, such as *Atopobium*, *Gardenella*, *Mobiluncus*, *Prevotella*, *Anaerococcus*, and *Sneathia*, are often associated with a symptomatic disorder of the vaginal microbiome known as bacterial vaginosis (BV) (Schellenberg et al., 2011; Ma et al., 2012).

The microbiome is regulated through (1) the microbial species that exert their own influence on species pressure, (2) the introduction of new species through foreign introduction, and (3) host factors such as genetics, diet, and hormones that promote the growth of certain bacterial genera. There have been a great deal of association studies performed analyzing the link between the vaginal microbiome and FGT barrier function and immunity, but studies of regulatory mechanisms have been lacking. In order to examine these relationships in depth, several models have been generated in which the conditions of both the lower and upper FGT are simulated and microbes of interest can be included to characterize their role in affecting immune response, barrier permeability, and the secretion of metabolites. These models present the best opportunity to move many of the discovered relationships past association and into causal explanation.

## Study Models of FGT Barrier Function

**Figure 2:**
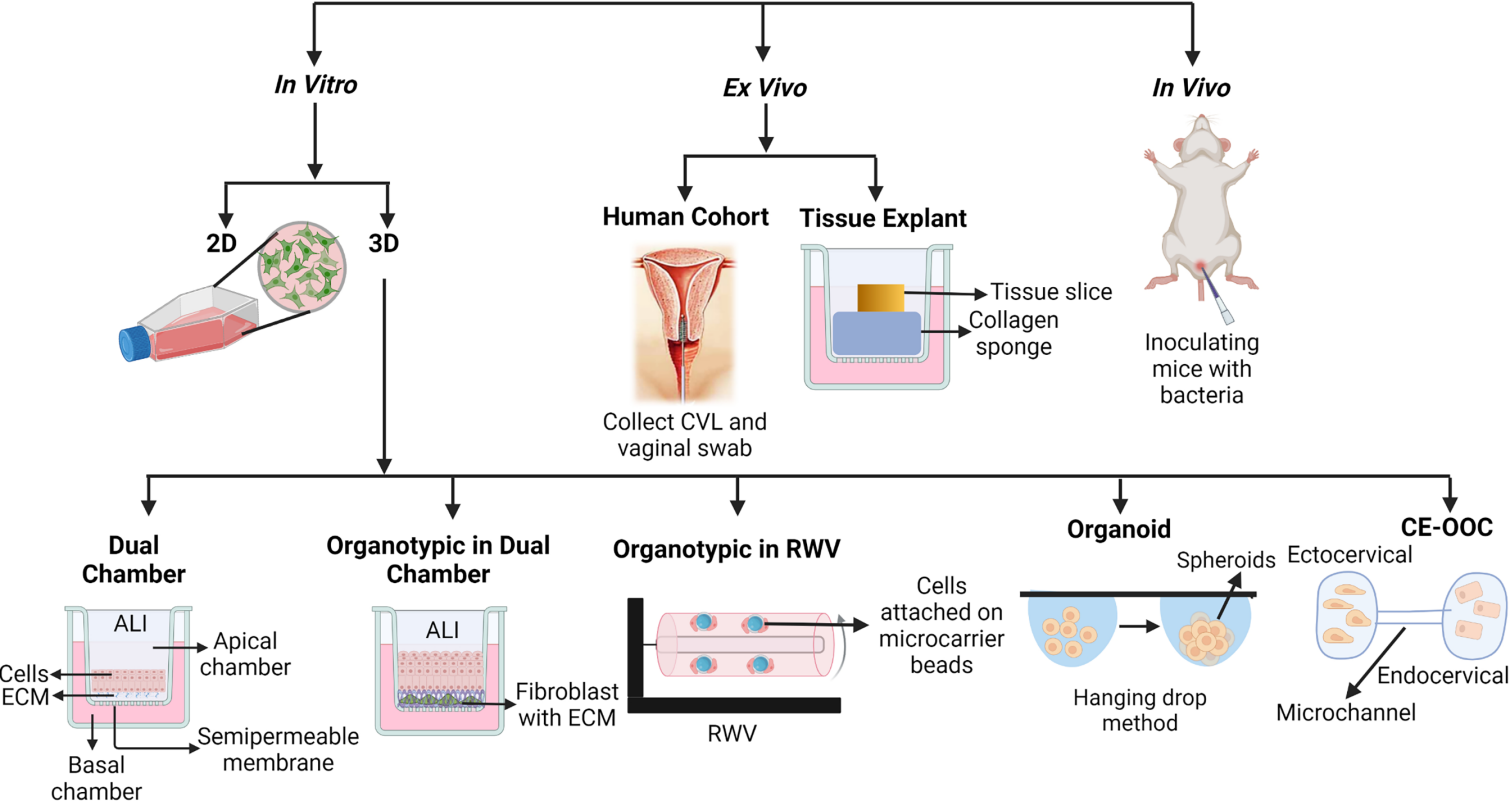
Different types of models to study cervicovaginal mucosal barrier function. 2D, Two dimensional; 3D, Three dimensional; ECM, Extracellular matrix; ALI, Air-liquid interface; CVL, Cervicovaginal liquid; RWV, Rotating well vessel; CE-OOC, Organ-on-chip cervical epithelial.

Most experimental models of the FGT mucosa focus primarily on the lower FGT, composed of the ectocervix and vagina, with a smaller number focusing on the upper FGT, composed of the endocervix and endometrium. Initial studies utilized monolayer cultures of cervical and vaginal epithelial cells to study barrier function, but more recently three-dimensional (3D) models using cervical and vaginal epithelial cells have been developed. Cervical and vaginal explant tissue models, and *in vivo* systems, have also been used to study the regulation of cervicovaginal barrier function. A brief description of these models, and their use in studying FGT mucosal barrier function, will be discussed in the following sections (**Figure 2** and **Table 1**).

**Table 1:** Comparison between different models used to study cervicovaginal mucosal barrier function.

Models	Brief description	Cells used	Applications	Advantages	Limitations	Reference
***In Vitro***	Monolayer (2D): Immortal cell lines are grown on tissue culture-treated plasticware	Vaginal (Vk2), ectocervical (Ect1), and endocervical (End1)	Wound healing, changes in the expression of transcripts, and secreted and intracellular proteins with various stimulation conditions	Fast, easy, and cost-effective	Cells are not differentiated, less (or no) cell to cell junction formation, and some proteins/molecules found in physiological tissues are not expressed when cells are grown in 2D	(Fichorova et al., 1997; Dusio et al., 2011)
	Dual-Chamber (3D): Cells are grown on a semipermeable membrane coated with ECM. Cells are grown using an ALI condition. Both immortal and primary cells are used in this model	Vk2 and primary genital epithelial cells (GECs)	Cell permeability, regulation of cell to cell junction formation and cell differentiation, and transmigration of pathogens or immune cells through the epithelial layer	Similar to physiological tissue in terms of multilayer formation, differentiation, and cell to cell junction formation of epithelial cells	High technical variability, large-scale experimental requirements, highly expensive, gene expression profiles are different between transformed and primary cells, mucus secretion is not reported, and it is not possible to study a mucosal anaerobic environment	(Gali et al., 2010; Kaushic et al., 2011; Lee et al., 2016)
	Organotypic model (3D) in RWV: Immortal epithelial cell lines are attached to collagen-coated microcarrier beads. Bead attached cells are transferred to the RWV bioreactor and cultured for 39-42 days	Vaginal (V191), endocervical (A2EN), and endometrial (HEC-1A)	Colonization effects of *Lactobacillus* species and BV-associated bacteria such as *Atopobium*, *Prevotella*, and *Gardenella* on the barrier function of epithelial cells	Fully differentiated epithelial cells with tight junctions, microvilli, and mucus secretion	High technical variability, large-scale experimental requirements, highly expensive, gene expression profiles are different between transformed and primary cells	(Laniewski et al., 2017; Łaniewski and Herbst-Kralovetz, 2019)
	Organotypic model (3D) in Dual Chamber: Primary epithelial cells are grown on a semipermeable membrane coated with an ECM and fibroblast cell mixture using a dual-chamber system. Cells are cultured in ALI conditions with nutrient supplements and growth factors. The model contains an apical cell layer, suprabasal layer, and basal cell layer after differentiation	Primary ectocervical cells isolated from vaginal-ectocervical (VEC) tissue	Effect of douching, feminine products, and anti-fungal creams on cervicovaginal epithelial cells	Express cytokeratins similarly to cervical tissue	High technical variability, large-scale experiment requirements, highly expensive, time-consuming, requires ethics approval for collecting primary tissue, mucus secretion is not reported, and it is not possible to study a mucosal anaerobic environment	(Aldunate et al., 2015)
	Stratified ectocervical organoid and cystic endocervical organoid models (3D): Cells are embedded into a basement membrane extract and plated in 30 µL droplets on pre-warmed 24-well suspension culture plates, then grown in culture media with growth supplements to develop spherical organoids	Primary ectocervical and endocervical cells from hysterectomy tissue	Study morphological, transcriptomic, and phenotypic differences of ectocervical and endocervical tissue, and infection studies with pathogenic microbes such as HSV2 and HPV	Possible to expand for longer passage numbers, and cryopreserved samples can be successfully used from thawed organoids		(Lõhmussaar et al., 2021)
	Organ-on-chip cervical epithelial (CE-OOC): Ectocervical and endocervical cells are grown in two different chambers which are connected with microchannels	Immortalized ectocervical and endocervical epithelial cells	Study EMT and MET processes of ectocervical and endocervical cells, and modulation of both ectocervical and endocervical cell function concomitantly in the same model in the presence and absence of infection and inflammation	Possible to study the nature and interaction of ectocervical and endocervical cells	Differentiation and tight junction formation of both ectocervical and endocervical cells, mucus secretion, and multilayer formation of ectocervical cells, are not reported, gene expression profiles are different in transformed and primary cells and it is not possible to study a mucosal anaerobic environment	(Tantengco et al., 2021)
***Ex Vivo***	Explant tissue: Cervical and vaginal tissue collected from human participants and cultured in ALI conditions in the dual-chamber system	Cervical or vaginal biopsy	Thickness of the epithelial layer, tissue permeability, tight junctions, cell proliferation, pathogen migration, regulation of protein and transcript expression, and the interaction between epithelial and fibroblast cells	The closest model to an *in vivo* system, since all cells in the explant model have physiological features	Highly expensive, the requirement for ethics approval, laborious, time-consuming, high biological variability, and it is not possible to study a mucosal anaerobic environment	(Phalguni et al., 2006; Mitchell et al., 2014; Goldfien et al., 2015)
	Human cohort: Establish a cross-sectional longitudinal cohort to collect CVL, epithelial cells, and/or immune cells	N/A	Study secreted proteins, secreted metabolites, mucus properties in CVL, and cellular proteins from epithelial cells	Ability to look at the impact of microbial diversity, demographic characteristics, individual behavior, etc., in affecting metabolites, mucus properties, and protein secretion and expression in relation to FGT barrier function	Highly expensive, the requirement for ethics approval, laborious, time-consuming, and challenges studying mechanism with a general restriction to observational findings	(Borgdorff et al., 2016)
***In Vivo***	Inoculating mouse models with commensal and BV associated bacteria present in the human FGT mucosa	N/A	Study secreted proteins, secreted metabolites, mucus properties in CVL, and cellular proteins from epithelial cells	Possible to study mucosal degradation of BV associated bacteria	Translation of findings to humans, expensive, requirement for ethics approval, laborious, and time-consuming	(Lewis et al., 2013)

ECM, Extracellular matrix; ALI, Air-liquid interface; GECs, Primary genital epithelial cells; RWV, Rotating well vessel; EMT, Epithelial-mesenchymal transition; MET, Mesenchymal-epithelial transition; CVL, Cervicovaginal lavage; FGT, Female genital tract; BV, Bacterial vaginosis; N/A, Not Applicable.

### *In Vitro* Models

Immortalized vaginal (Vk2), ectocervical (Ect1), and endocervical (End1) epithelial cell lines have been generated to study FGT epithelial function by transducing the primary cells isolated from cervicovaginal biopsies with human papillomavirus 16 (HPV-16) E6 and E7 genes (Fichorova et al., 1997). These epithelial cell lines have been used in generating both 2D (monolayer) and 3D models (**Figure 2**). These epithelial cells are grown on tissue culture-treated plasticware in monolayer models to study the effects of exogenous factors on epithelial function and properties. Vk2, Ect1, and End1 cells have been reported to express certain toll like receptors (TLRs) in 2D culture and have been exposed to TLR agonists to induce the production of cytokines, chemokines, and antimicrobial peptides (AMPs) (Fichorova et al., 1997; Dusio et al., 2011). Study of epithelial wound-healing properties has primarily been performed using 2D culture models (Dusio et al., 2011). The FGT mucosal metabolite low-molecular-weight hyaluronic acid (LMW-HA) has been shown to regulate barrier function using 2D culture of Vk2 cells. Treatment of Vk2 monolayers with LMW-HA induces secretion of AMPs, such as β-defensin-2, and promotes the wound-healing process by increasing migration of Vk2 cells through activation of phosphatidylinositol 3-kinase and myosin light chain kinase (Dusio et al., 2011). The 2D epithelial monolayer model is often used because of its simplicity, short-time frame, cost-effectiveness, and reproducibility of the results. The 2D monolayer model of cervicovaginal epithelium, however, differs vastly from physiological cervicovaginal tissue in terms of cellular differentiation, proliferation, protein expression, multilayer formation, and cell to cell junction formation between stratified squamous epithelial cells, as well as in the interaction between epithelial cells and the extracellular matrix (ECM) which is completely absent in the 2D model (Soares et al., 2012; Chitcholtan et al., 2013) (**Table 1**). Hence, 3D models of the cervicovaginal epithelium have been developed to overcome some of these limitations.

In 3D models, epithelial cells are grown on ECM such as collagen, matrigel, or fibronectin. When a dual-chamber culture system is used in a 3D model, transwell inserts with semipermeable membranes coated with ECM material are used (Gali et al., 2010; Lee et al., 2016; Woods et al., 2021). Primary or immortalized epithelial cells are grown on the supporting ECM in the upper chamber, and the lower chamber can be used for culturing any tissue resident cells, such as immune cells. Using this culture system, epithelial cells can be induced to differentiate and polarize in air-liquid interface (ALI) conditions (Lee et al., 2016). In ALI conditions, liquid culture media in the upper chamber (the apical side of the epithelium) is removed after the epithelial monolayer on the ECM becomes confluent (**Figure 2**), allowing the nutrients to be supplied by the culture media in the bottom chamber (the basal side of the epithelium). ALI conditions, simulating a mucosal environment where nutrients are supplied by blood and epithelium is exposed to air, trigger cellular differentiation and formation of cell-cell junctions (Lee et al., 2016). This model is suitable for studying epithelial integrity, regulation of tight junction and adhesion junction formation, and transmigration of pathogens through the epithelium. Trans-epithelial electrical resistance (TEER) and fluorophore-conjugated dextran diffusion assay are commonly used to assess epithelial integrity in the dual-chamber model systems (Gali et al., 2010). Vk2 and Ect1 cells in particular have been grown in dual-chamber 3D culture model systems to study epithelial immune and barrier function (Gali et al., 2010; Lee et al., 2016) (**Table 1**). The ALI model of Vk2 cells exhibits both multilayer and tight junction formation, and has been used to show that stimulation of Vk2 cells with the hormone progesterone increases their susceptibility to herpes simplex virus 2 (HSV2) infection (Lee et al., 2016). Primary genital epithelial cells (GECs), particularly endocervical and endometrial cells, have been isolated from hysterectomy tissue, cultured in the dual-chamber system, and shown to form tight junctions as well (Kaushic et al., 2011; Ferreira et al., 2013). Following exposure to HIV-1, increased GEC permeability was observed in this model system. Co-culture of HIV-exposed GECs with the probiotic bacteria *Lactobacillus reuteri* RC-14 and *Lactobacillus rhamnosus* GR-1, as well as the female sex hormone estrogen, was shown to restore GEC barrier function and tight junction formation (Dizzell et al., 2019). The major limitation of this 3D model includes high technical variability, variations between cell lines and primary cells, and, most of all, its deviation from physiological conditions, for example mucus-secretion and an anaerobic environment (Gali et al., 2010; Lee et al., 2016). As a consequence, this is not an ideal system to study interactions between the microbiome and epithelium.

The organotypic vaginal-ectocervical (VEC) tissue model also uses a dual-chamber system. In the VEC tissue model, primary ectocervical cells are isolated from the VEC tissue obtained from hysterectomy and cultured on transwell inserts using ALI conditions to induce differentiation and polarization (Aldunate et al., 2015) (**Figure 2**). Before culturing the ectocervical epithelial cells on the transwell inserts, the inserts are seeded with a fibroblast and collagen mixture to create a basal membrane. Fibroblast cells are isolated from the same donor used to obtain ectocervical cells to avoid allogeneic immune effects. Ectocervical cells are then cultured on top of the fibroblast and collagen gel matrix, which creates a VEC tissue model containing an apical cell layer, suprabasal layer, and basal cell layer post-differentiation, along with a similar type of cytokeratin expression compared to actual VEC tissue (Ayehunie et al., 2006). This model shows more resistance against washings, lubricants, and anti-fungal creams compared to non-organotypic epithelial models, and represents a more relevant physiological scenario (Aldunate et al., 2015) (**Table 1**). Although the 3D and VEC tissue dual-chamber models are advantageous for cell differentiation, tight junction formation, and cytokeratin expression, they still lack many aspects of the FGT mucosa. For example, studying the barrier properties of mucus at the FGT mucosa is not possible with these models, since mucus secretion has yet to be reported using VEC tissue models.

Another 3D epithelial tissue model using a rotating wall vessel (RWV) bioreactor and immortal vaginal (V191), endocervical (A2EN), and endometrial (HEC-1A) cell lines has been developed by Dr. M. Herbst-Kralovetz’s group. The epithelial tissue in this model has been shown to secrete mucus, and exhibit proper cytokeratin expression, microvilli, and tight junction formation (**Figure 2** and **Table 1**) (Hjelm et al., 2010; Radtke et al., 2012; Doerflinger et al., 2014; Laniewski et al., 2017; Łaniewski and Herbst-Kralovetz, 2019; Ilhan et al., 2020). To start, epithelial cells are grown on collagen-coated dextran microcarrier beads. The cell and bead suspensions are then transferred to the RWV bioreactor to be cultured for 39-42 days to generate fully differentiated 3D tissue models (**Figure 2**). When used in studying the interaction between epithelium and microbes, colonization of this vaginal RWV-3D tissue model with the anaerobic bacterium *Atopobium vaginae* resulted in increased secretion of cell-associated mucins, proinflammatory cytokines and chemokines, and AMPs as compared to colonization with *Lactobacillus* species (Doerflinger et al., 2014). Although the RWV-3D tissue model allows the evaluation of the role of mucus and anaerobic microbes in regulating epithelial barrier function, the challenges of reproducibility and the differences in gene expression between cell lines and primary tissue have not been resolved.

The organoid models, such as the stratified ectocervical organoid and cystic endocervical organoid models, use primary ectocervical and endocervical cells isolated from cervical tissue obtained during hysterectomy procedures (**Table 1**) (Lõhmussaar et al., 2021). Primary cells are embedded into a basement membrane extract and plated in 30 µL droplets on pre-warmed 24-well suspension culture plates, then grown in culture media with growth supplements to develop spherical organoids (**Figure 2**) (Lõhmussaar et al., 2021). Both organoid models have been used to study cellular susceptibility to HSV-2 infection, but have yet to be used to study epithelial barrier function (Lõhmussaar et al., 2021).

The most current *in vitro* cervical tissue model is the organ-on-chip cervical epithelial (CE-OOC) model. The CE-OOC model contains two chambers to co-culture immortalized ectocervical and endocervical epithelial cells, and the two chambers are connected by micro-channels that allow the epithelial cells to migrate (**Figure 2**) (Tantengco et al., 2021). The phenomena of epithelial to mesenchymal transition (EMT) and mesenchymal to epithelial transition (MET) have been studied using the CE-OOC model (**Table 1**). In that, ectocervical cells have been reported to form fibroblastoid morphology, and endocervical cells form pseudopods while migrating through micro-channels from one chamber to the other, in monoculture systems (Tantengco et al., 2021). This model has been used to study normal and pathogenic cellular remodeling of the cervix, and the interaction between ectocervical and endocervical cells during infection and inflammation (Tantengco et al., 2021). Although *in vitro* 3D models of epithelium provide a close resemblance to physiological tissue, they still present challenges, such as high technical variability, large-scale experimental requirements, high costs, and being time intensive. Moreover, these 3D models cannot be used to study epithelial barrier properties in the presence of non-epithelial cells such as stromal and sub-epithelial immune cells (**Table 1**).

### *Ex Vivo* Models

Cervical tissue explants, either collected from cervical tissue after hysterectomy or cervical punch biopsies, have been previously used for HIV transmission studies (Phalguni et al., 2006; Grivel and Margolis, 2009; Mitchell et al., 2014; Trifonova et al., 2018). Ectocervical, endocervical, and transformation zone physiology in particular have been studied using this model (**Table 1**) (Goldfien et al., 2015). The cervical explant tissue model is the closest model to an *in vivo* model, since the explant tissue is composed of epithelial cells, subepithelial immune cells, and fibroblasts. This model allows for studying the interaction between epithelial, immune, and fibroblast cells during infection as well. Fresh and cryopreserved cervical tissue explants provide similar results in infection studies, indicating the ability to use frozen tissue explants as an alternative to fresh tissue explants. This allows researchers to circumvent one of the major potential drawbacks of using cervical tissue explants—sample preservation (Phalguni et al., 2006; Fox et al., 2017). The cervical and endometrial tissue explant model has been used to observe the effect of hormonal contraceptives in changing barrier properties and infection susceptibility as well. Explant tissue models are used to study hormonal contraceptive effects on epithelial layer thickness, cell proliferation, alteration of tight junction and secretory proteins, and gene transcription (**Table 1**). The explant tissue model is not without challenges. It still lacks an anaerobic environment, and can be highly variable due to biological differences between tissue donors.

Besides isolating explant tissue, cervicovaginal lavage (CVL) and vaginal swabs from women have also provided crucial insights in uncovering the regulation of cervicovaginal barrier function (**Figure 2**). Proteomic studies with CVL using high throughput mass spectrophotometry have reported that an increase in vaginal microbiome diversity is associated with a decrease in a protein expression profile that supports barrier function (Borgdorff et al., 2016). The limitations that come with this model are challenges achieving the proper ethics and appropriate number of clinical participants, along with longer time frames and higher expense (**Table 1**).

### *In Vivo* Models

Although not perfect, the use of animal models allows the investigation of how vaginal bacterial composition, and other local factors, regulate cervicovaginal barrier function in a complete living system (Lewis et al., 2013). Various types of tissue, such as upper and lower FGT, and fluids, such as CVL and plasma, can be isolated from animal models for systematic analyses to obtain a more comprehensive, and unbiased, picture (**Figure 2** and **Table 1**). For example, a study using C57BL/6 mice (6–8 weeks old) colonized with the mouse-adapted strain of *G. vaginalis* showed degradation of vaginal mucus and consequent weakening of vaginal barrier properties compared to control mice (Lewis et al., 2013). To study microbes that infect only human cells, bone marrow-liver-thymus (BLT)-transplanted humanized mouse models have been used. The BLT mouse has been used to test microbicides against HIV-1 and study changes in cervicovaginal barrier properties during HIV transmission (Paul et al., 2011; Maud and Andrew, 2013; Nirk et al., 2018; Shariq et al., 2019). These BLT mouse models, in concert with non-human primate (NHP) models, are critical in studying the interaction between cervicovaginal epithelial cells and sub-epithelial immune cells in the regulation of cervicovaginal barrier function in the context of HIV infection. The microbial composition and physiological conditions of these animal models are different from that of the human, and need to be considered when translating findings to human research or treatment.

## Factors That Affect/Regulate Epithelial Barrier Function

### Microbiome Factors

#### Microbial Diversity

The composition of vaginal microbial communities influences the vaginal defense system of the mucosal barrier by inhibiting the growth of disease-causing pathogenic microorganisms by either producing microbicidal compounds, or by enhancing host physical and immunological barrier function. One of the critical components of physical barrier function at the FGT mucosa is mucus. *L. crispatus* dominated vaginal microbiomes are associated with mucus that traps microbes, such as HIV, more efficiently as compared to the mucus associated with vaginal microbiomes dominated by *L. iners* or *Gardenella* species (Nunn et al., 2015). The knowledge of how vaginal microbes affect the role of mucus in defense remains scarce. Mucus can be compromised in the case of degradation by BV-associated bacteria. BV-associated bacteria, such as *Gardnerella*, *Prevotella*, and *Bacteroides*, make the sialidase enzyme that actively degrades sialic acid—a critical component of FGT mucus (Lewis et al., 2013; Vagios and Mitchell, 2021). Other mucus-degrading enzymes, including mucinases, sulfatases, galactosidases, and prolidases, damage FGT barrier integrity by contributing to the watery vaginal discharge characteristic of BV (Lewis et al., 2013; Vagios and Mitchell, 2021). Besides mucus, host proteins in vaginal secretions also play a key role in vaginal epithelial barrier function. Proteomic analysis of CVL samples collected from a cross-sectional study of 50 Rwandan female sex workers resulted in four groups with distinct compositions of dominating vaginal microbiomes. Group 1 is associated with *L. crispatus* dominant vaginal microbiomes, group 2 is associated with *L. iners* dominant vaginal microbiomes, group 3 is associated with anaerobe dominant vaginal microbiomes along with intermediate vaginal microbial diversity, and group 4 is associated with anaerobe dominated vaginal microbiomes along with high vaginal microbial diversity. The protein profiles of the CVL samples from these women further showed that increasing vaginal microbial diversity was positively associated with increased protein levels in the biological pathways of cell death, proteasome and protease activity, and pro-inflammatory cytokines. The increased vaginal microbial diversity was negatively associated with the levels of keratin protein, cornified envelop proteins, humoral immune molecules, such as IGHA1 and IGHG2, and anti-protease activity (Borgdorff et al., 2016). Together, this study showed that increasing vaginal microbial diversity is associated with increases of detrimental, and decreases of beneficial, host protein levels pertaining to epithelial vaginal barrier function. As these are associations, it remains to be shown whether changes in host barrier function permit the diversification of vaginal microbiome or whether the diversification of vaginal microbiome causes the changes in host barrier function. Moreover, the molecular mechanisms of how vaginal microbial diversity interacts with vaginal mucosal protein expression remain to be sought.

AMPs are part of the mucosal chemical barrier against the foreign environment. Vaginal levels of AMPs are also found to be closely related to vaginal microbial diversity. Increased FGT microbial diversity is associated with decreases in AMPs, such as lysozyme C (LYZ) and ubiquitin (RPS27A), and increased S100A9 (Borgdorff et al., 2016). Changes in AMP levels have also been reported during BV, with human beta defensin-2 (HBD-2), lactoferrin, and cathelicidin (LL-37) found to be higher in the CVL of women with BV (Fan et al., 2008; Gregory et al., 2011; Frew et al., 2014). *In vitro* experiments also found increased secretion of HBD-2 from epithelial cells co-cultured with BV associated bacteria, such as *A. vaginae* and *L. iners* (Doerflinger et al., 2014). However, how increases in vaginal microbial diversity, and the condition of BV, influence secretion of AMPs remains to be explored.

#### Microbial Byproducts

Vaginal fluid contains not only host factors, but also microbial metabolites and enzymes. This profile is largely dependent on the composition of the vaginal microbiome and the interaction between host cells and microbiota (Srinivasan et al., 2015). The D-lactic acid (D-LA) isoform is predominantly produced over L-lactic acid (L-LA) in *L. crispatus*, *L. gasseri*, and *L. jensenii*, as compared to *L. ine*rs, which produces only L-LA (Steven et al., 2013; Nunn et al., 2015). It is suggested that vaginal concentrations of D-LA and L-LA may be a key determinant in vaginal microbiome diversity and vaginal health. Vaginal mucus with high concentrations of D-LA exhibits enhanced trapping of HIV-1 particles, compared to mucus with low concentrations of D-LA characteristic of *L. iners* dominant microbiomes. (Nunn et al., 2015). Both D-LA and L-LA can reduce the pH of the vaginal mucosa and prevent the growth of yeast and other microbes, as well as reduce the release of pro-inflammatory molecules by cervicovaginal epithelial cells (Hearps et al., 2017). Hearps et al. showed that *in vitro* treatment of human vaginal and cervical epithelial cell lines with LA (pH 3.9) induced production of the anti-inflammatory cytokine IL-1RA. Further, when added simultaneously or prior to stimulation, LA inhibited the TLR agonist-induced production of pro-inflammatory mediators IL-6, IL-8, TNFα, RANTES, and MIP3α from epithelial cell lines, and prevented IL-6 and IL-8 production elicited by exposure to seminal plasma (Hearps et al., 2017). Similar anti-inflammatory effects of LA hold true for primary cervicovaginal cells and when organotypic epithelial tissue models are used.

In addition, *Lactobacillus* species, except for *L. iners*, also produce hydrogen peroxide and bacteriocins as byproducts that inhibit growth of pathogenic non-indigenous bacteria (Vallor et al., 2001; Mich` et al., 2015). Alternatively, *L. iners* secretes cholesterol-dependent cytolysin (CDC) and *G. vaginalis* secretes vaginolysin, both of which are known to have cytotoxic activity against vaginal and cervical epithelial cells (Cadieux et al., 2009; Macklaim et al., 2013). In an *in vitro* study, culture supernatant of *L. iners* and *Gardenella* increased the permeability of ectocervical and endocervical cell culture by impairing the cellular adhesion molecule E-cadherin, and increased release of soluble cadherin compared to *L. crispatus* culture supernatant (Anton et al., 2018). In contrast, *L. crispatus* secreted byproducts in culture supernatant that helped to restore ectocervical and endocervival barrier integrity, previously disrupted by *Gardenella* culture supernatant (Anton et al., 2018). Unfortunately, this study did not explore the byproduct profiles in these culture supernatants. Succinate and short-chain fatty acids, such as butyrate, propionate, and acetate, commonly present in the gut mucosa, are also found at low levels in the vaginal mucosa. In the condition of BV, however, much higher levels of SCFAs have been found (Mirmonsef et al., 2011). A meta-transcriptomic study also showed the up regulation of butyrate metabolizing enzymes, butyryl-CoA-dehydrogenase, and butyrate kinase during BV, associated with increased *P. amnii* and *Megasphaera* (Macklaim et al., 2013). Usually, SCFAs exhibit a beneficial role in gut mucosa by improving gut epithelial integrity, differentiation, proliferation, and reducing the secretion of pro-inflammatory molecules from gut mucosal epithelial and immune cells (Blacher et al., 2017). Knowledge of the role of SCFAs in the FGT mucosa remains limited. An *in vitro* study found contrary results that high levels of SCFAs can induce pro-inflammatory molecule secretion from vaginal and cervical epithelial cells (Delgado-diaz et al., 2020). More studies are required to explore the impact of higher SCFA levels in changing FGT mucosal barrier function and its association with BV conditions.

### Host Factors

#### Immune Cells/Immune Factors

The description of the FGT immunologic milieu deserves another review or book chapter by itself. This review will only summarize key immune cells and mediators that are relevant for the discussion of the interactions between FGT host cells and the microbiome.

The lower FGT is characterized by IgG present from vaginal transudate and local mucosal IgA antibodies (Fahrbach et al., 2013; Gunn et al., 2016), antimicrobial peptide secretion (Yarbrough et al., 2015), and the presence of both adaptive and innate immune cells (Zhou et al., 2018; Mei et al., 2019). T cells exist primarily below the substratum of the epithelium, and provide cytokine and chemokine responses to address invading pathogens or barrier tissue damage. Macrophage and neutrophils are present in the tissue, maintaining the homeostasis between the microbial environment and the host epithelium. In response to invasion of the FGT, macrophage and neutrophils function to remove pathogenic, and bystander commensal, bacteria and promote wound healing (Wira et al., 2005; Jennifer et al., 2021). The FGT immunologic milieu changes as the FGT transforms to the upper compartment, where the physiological emphasis changes from focusing on homeostasis and defense to focusing on the balances of both homeostasis and the readiness to maintain a potential pregnancy (Ochiel et al., 2008; Agostinis et al., 2019). The uterine cavity is home to the unique uterine natural killer (uNK) cell population, as well as a high degree of gamma delta (γδ) T cells and macrophage (Amy et al., 2012; Zhou et al., 2018; Monin et al., 2020). At the upper FGT, the profiles of secreted antimicrobial peptides and cytokines focus on the production of anti-inflammatory cytokines alongside AMPs. The uterus is able to achieve high degrees of immune surveillance due to the presence of lymphoid aggregates, dense groupings of B and CD8^+^ T cells, which allow for targeted and controlled responses to foreign antigen in the localized immune environment of the uterus (Yeaman et al., 2001; Agostinis et al., 2019). Advances in uterine immune regulation are summarized in detail in the review articles by Anne et al, Abebe et al, and Liman et al. (Anne et al., 2014; Abebe et al., 2021; Liman et al., 2021).

The composition of the microbiome found in the lower and upper FGTs are quite distinct; however, there is a clear association between the species present within the lower FGT and the species found in the upper FGT (Baker et al., 2018; Agostinis et al., 2019). For example, *Lactobacillus* species typically dominate the lower FGT, but comprise only approximately a quarter of the upper FGT microbiome on average, while other genera such as *Pseudomonas*, *Acinetobacter*, and *Sphingobium* make up the remaining portion. It is unknown whether these genera arise from occult introduction, or if they travel from the gut and other sites to colonize the uterus directly (Łaniewski et al., 2020). For much of the history of FGT microbiome research, the upper FGT has been thought to be a sterile environment in healthy women, but this notion has been challenged by recent advances in sequencing and sampling technology that have uncovered a potentially omnipresent, if variable, uterine microbiome.

Microbiome composition has been associated directly with health and disease (Swain and Ewald, 2018). The interplay between the microbiota and the human immune system in the gut, and to a lesser extent in the respiratory tract, have been examined extensively during the last decade to yield translational knowledge with implications for mucosal health. However, such knowledge at the FGT remains scarce. It was thought that host immune cells and immune mediators play a direct role in shaping the composition of the FGT microbiome, which consequently impacts barrier function and the ability to protect against STIs. There still remains no direct evidence for this hypothesis, however. Based on what has been learned of the gut mucosa (Yasmine and Oliver, 2017; Zheng et al., 2020), the FGT microbiota could provide critical signals for the development and function of the host immune system, and, the host immune system, in turn, could have evolved multiple means by which to maintain its symbiotic relationship with the microbiome. The microbiome of the lower and upper FGTs interact with the immune mechanisms of the host primarily through interaction with immune components, such as immunoglobulins, where they can reduce the efficacy of these molecules *via* neutralization through degradation or competitive binding. General inflammation and mucus breakdown has also been observed with the CST-IV, or BV-associated, microbiome (Lewis et al., 2013; Lacroix et al., 2020). How the host immune system is ‘educated’ to accommodate the commensal microbial community is critical. At the gut mucosa, such mechanisms exist to tolerate food antigens and microbes that are essential for gut function (Chistiakov et al., 2014). If such a tolerating mechanism fails, undesirable inflammation results in disease. At the FGT, the combination of liquefaction of the mucus barrier and increased recruitment of immune cells due to local pro-inflammatory cytokines and chemokines leads to increased risk of immune cell infection by pathogens such as HIV, as well as direct epithelial infection by pathogens such as HSV-2 and chlamydia (Koumans et al., 2007; Passmore et al., 2016; Bautista et al., 2017). This also creates a self-reinforcing cycle where host pro-inflammatory immunity can have non-specific and antigen-specific removal and/or inhibition of commensal microbes, and potentially further prevent the establishment of a *Lactobacillus* dominant microbiome. Disrupting the homeostasis of the commensal microbial environment may potentiate further exacerbation of inflammation at the FGT mucosa. How the uterus, and pregnancy benefit from hosting commensal microbes, and how the uterine/endometrial immune system tolerates its associated microbiota to maintain a homeostatic microenvironment, urgently require further studies.

#### Hormones and Age

Hormones have a direct influence on both the immune system and the microbial community present within the FGT. Together, all three play crucial roles in regulating FGT barrier function. During pregnancy, and at puberty, increases in the female sex hormone estrogen promote the proliferation and differentiation of vaginal epithelial cells. Glycogen from exfoliated and lysed epithelial cells is converted by α-amylase, and the product is then metabolized to lactic acid by *Lactobacillus* species in the vaginal lumen. A glycogen-rich environment directly promotes the growth and dominance of *Lactobacillus* species (Amabebe and Anumba, 2018). This is further reinforced through the production of hydrogen peroxide, bacteriocins, and biosurfactants from *Lactobacillus* species. Elevated estrogen levels are also associated with a thicker epithelial lining with greater elasticity, perhaps *via* promoting the proliferation and differentiation of FGT epithelial cells. The synergistic effect of estrogen-high and *Lactobacillus* dominant environments associates most strongly with improved barrier function, vaginal health metrics, and protection from STIs and cancer.

In contrast, progesterone, the other commonly considered female sex hormone, has no significant association with any CST group. Although progesterone has been shown to play a protective role in preventing inflammation-induced preterm labor during pregnancy (Dodd and Crowther, 2009), the use of the hormonal contraceptive medroxyprogesterone acetate, a progesterone analogue that has different biological effects, is associated with increased vaginal microbiome diversity that potentially modulates vaginal inflammation and increased HIV-1 susceptibility in humanized mouse models (Wessels et al., 2019). The use of depot medroxyprogesterone acetate (DMPA) is also associated with the CST IV-type microbiome in the FGT, and a higher risk for acquiring BV (Wessels et al., 2019). The reduction in robust epithelial barrier support, with concomitant increased risk of inflammatory and dysbiotic states, is thought to be partially responsible for explaining DMPA’s association with increased HIV risk and proteome signatures of epithelial barrier disruption (Birse et al., 2017). Several studies also reported the association of elevated progesterone with a more inflammatory environment and a less robust barrier at the vaginal epithelium *via* unknown mechanisms (Tjernlund et al., 2015; Vitali et al., 2017; Bradley et al., 2018). While the mechanism requires further investigation, DMPA has also been reported to have neutral or even detrimental effects to the elasticity of the vaginal membrane leading to increased chances of vaginal dryness and epithelial tearing (Irvin and Herold, 2015; Birse et al., 2017; Zalenskaya et al., 2018). With the cyclic nature of the human menstrual cycle, FGT barrier function may change throughout a given cycle depending on the balance of estrogen and progesterone. Indeed, during menses there is often a complete loss of a woman’s characteristic CST, which is often restored shortly at the end of menses (Gajer et al., 2012). In general, the human FGT microbiome appears to remain stable despite the shifts in hormones throughout the menstrual cycle. While it is clear that highly elevated estrogen and DMPA levels have direct effects on vaginal microbiome composition, it is unknown if the FGT microbiome has its own impacts on hormone production, or activity, locally within the FGT.

Outside of female sex hormones, oxytocin, a hormone and neuropeptide normally produced in the hypothalamus and released by the posterior pituitary, has been explored as an alternative to estrogen-based therapies for vaginal dryness. It has been shown to be effective clinically as a vaginal gel in alleviating menopausal vaginal symptoms and *in vitro* in promoting proliferation of Vk2 vaginal epithelial cell lines (Kallak and Uvnäs-Moberg, 2017; Torky et al., 2018). Tremendous work remains to validate oxytocin’s role in the interplay between the immune system, the microbiome, and its effects on FGT barrier function.

Age, inextricably linked with hormone levels, is a core determinant of the FGT microbiome. Data across ages for FGT microbiomes only exists for the lower FGT, with microbiome signatures for the upper FGT only existing for women of reproductive age (Gupta et al., 2019; O’Callaghan et al., 2020). In early infancy the lower FGT is colonized by *Lactobacillus* species due to the prenatally inherited high estrogen levels from the mother’s blood. Approximately six weeks post-birth, vaginal microbiomes progress to a microbiome more reminiscent of skin with a complete loss of *Lactobacillus* dominance. This persists up until puberty, when production of estrogen increases, and menstruation begins, and *Lactobacillus* species dominate the lower FGT once again. This is notably true in those of Caucasian and Asian descent, but this pattern is less prevalent in those of African ancestry where vaginal microbiomes may develop into polymicrobial communities with greater frequency (Ravel et al., 2011; Fettweis et al., 2014). It remains unknown whether this is the influence of genetic or cultural and epigenetic factors. Regardless, the higher prevalence of polymicrobial vaginal microbiomes is considered a risk factor for HIV acquisition and BV development in this population (Kyongo et al., 2015; Alcendor, 2016; Dabee et al., 2019). Along with changes to the microbiome, pH levels begin to decrease from 7.0 in pre-puberty to the 4.0–5.0 range typical of normal, healthy, vaginas (Gupta et al., 2019). This is mostly due to the dominance of *Lactobacillus* species and the production of lactic acid and hydrogen peroxide at the vaginal mucosa. These influences persist throughout a woman’s sexual lifetime, even after the onset of menopause, where, due to a decline in estrogen levels the microbiome transitions back to a phenotype similar to the pre-pubertal state, with a coinciding increase in pH and loss of *Lactobacillus* dominance. This puts postmenopausal women at increased risk of barrier disruption and STIs due to the combined effects of a less robust epithelial barrier, a lack of *Lactobacillus*-mediated protection at the FGT, and increased pH levels (Brotman et al., 2018; Gliniewicz et al., 2019; Murphy et al., 2019).

#### Host Metabolites

It is not always possible to distinguish host vs microbial metabolites, and, indeed, both can be responsible for the production of the same molecule. Lactic acid, for example, is produced by both vaginal epithelial cells and by *Lactobacillus* species as a result of glycogen metabolism and is thought to be one of the main drivers behind the reduction in vaginal pH seen with increased *Lactobacillus* dominance (Amabebe and Anumba, 2018; Ratter et al., 2018; Song et al., 2020). There is evidence that in heightened states of inflammation, the metabolic profile of the FGT changes to one consistent with greater cellular leakage and cellular breakdown. In addition, variation in the metabolic profile of the FGT between the subsets of women with or without BV has been observed (Srinivasan et al., 2015; Parolin et al., 2018; Ceccarani et al., 2019). Lower levels of lactate, amino acids, and dipeptide, and higher amounts of bioactive metabolites such as signaling eicosanoid 12-hydroxyeicosatetraenoic acid (12-HETE), were found in the vaginal mucosa of women with BV (Srinivasan et al., 2015; Parolin et al., 2018). Lower amounts of sialic acid and higher levels of mannose epitopes were also found in the CVL of women with BV as compared to those without BV (Linlin et al., 2015). Here, decreased levels of vaginal sialic acid during BV may be explained by the increased activity of the sialidase enzyme produced by BV-associated bacteria (Lewis et al., 2013; Vagios and Mitchell, 2021). How changes in host metabolites impact microbial composition and host cell function, and how the microbiome, in turn, influences the metabolic expression of host cells remains to be characterized. It is still unclear to what degree host-derived metabolites originate from the blood, and enter the lower FGT *via* plasma transudate, and to what degree they are produced locally by resident epithelial and immune cells.

## Gaps in Knowledge

Our knowledge regarding the microbiome and host factors in regulating cervicovaginal mucosal barrier properties lags far behind what is known at other mucosal sites, such as the gut and respiratory mucosa. Although higher diversity of the vaginal microbiome is associated with reduced FGT barrier properties, the mechanism behind the feature of *Lactobacillus* species in preventing growth and biofilm formation of pathogenic microbes at the FGT remains a particularly interesting point of research (Parolin et al., 2021). Similarly, the interaction of host cells, including immune cells and non-immune cells, with bacterial communities in different CST groups and BV-associated microbes needs to be investigated in depth. Recently, studies showed the difference in the metabolomic profile of the vaginal mucosa in healthy and BV states (Srinivasan et al., 2015; Parolin et al., 2018; Ceccarani et al., 2019). However, the effect of specific bacteria in regulating host metabolic pathways, and the role of those differentially abundant metabolites in regulating barrier properties and disease susceptibility, has not yet been explored. Furthermore, tryptophan levels were reported to be higher in women’s vaginal mucosa during dysbiosis, or BV, as compared to healthy women. The effect of gut bacteria-derived tryptophan metabolites, such as indole-3-ethanol, indole-3-pyruvate, and the receptor of these metabolites, aryl hydrocarbon receptor (AHR), in regulating gut epithelial and skin keratinocyte barrier function implores us to investigate their role in vaginal epithelial barrier function (Scott et al., 2020; Uberoi et al., 2021). Metatranscriptomic, metaproteomic, and metabolomic approaches are necessary to identify reproductively and metabolically active bacteria, secreted bioactive metabolites from those bacteria, and metabolic proteins or enzymes involved in generating those metabolites. Altogether this can be used to determine the role of those bacteria and bioactive metabolites in regulating the ecology of the FGT mucosal microenvironment and host barrier properties (Macklaim et al., 2013). Besides having gaps in which factors affect FGT barrier function, using both *in vitro* and *ex vivo* models to test microbiome effects on FGT barrier function presents its own challenges, especially with regards to representing the physiological condition of the FGT mucosa properly. For example, studying the composition of microbes, microaerophilic and low pH conditions of the mucosa, and the shedding nature of the outer epithelial layer are all insufficient with current models. *In vivo* models provide alternative approaches, but the translation of the findings from animal models to human application is challenging given the discrepancy in microbial compositions.

## Summary

Mucosal barriers are critical gatekeepers in allowing nutrients and beneficial molecules to, and preventing pathogenic and infectious agents from, entering the body. The health of the FGT is guarded by its mucosal barriers and the integrity of FGT mucosal barriers is governed by the interplay between host immune system, hormones, and the FGT microbiota. However, our knowledge of how FGT homeostasis is maintained is still lacking. With this knowledge, an understanding of risk factors for STI acquisition, and a broader understanding of vaginal and uterine health, can be implemented in future research questions as well as care and policy decisions for sexual health, sexual quality of life, and reproductive interventions.

## Author Contributions

AP participated in the researching and reviewing of research articles, defining the hypothesis, writing and revising the manuscript, and designing **Figure 1**. ABS participated in the researching and reviewing of research articles, defining the hypothesis, writing and revising the manuscript, and designing **Figure 2** and **Table 1**. RCS participated in the researching and reviewing of research articles, defining the hypothesis, writing, reviewing, and revising the manuscript, and reviewing the figures and table. AP and ABS contributed equally to this work and share first authorship. All authors contributed to the article and approved the submitted version.

## Funding

This work is primarily funded by the National Microbiology Laboratories of Public Health Agency of Canada. AP, ABS and RCS are supported by Public Health Agency of Canada. RCS also received research funding from the Canadian Institute of Health Research (CIHR/NIH-154043).

## Conflict of Interest

The authors declare that the research was conducted in the absence of any commercial or financial relationships that could be construed as a potential conflict of interest.

## Publisher’s Note

All claims expressed in this article are solely those of the authors and do not necessarily represent those of their affiliated organizations, or those of the publisher, the editors and the reviewers. Any product that may be evaluated in this article, or claim that may be made by its manufacturer, is not guaranteed or endorsed by the publisher.
